# Integrated single-cell transcriptomic atlas of human gastric and colorectal tissues across diverse phenotypes

**DOI:** 10.1038/s41597-026-07108-3

**Published:** 2026-03-26

**Authors:** Yunjin Go, Aki Uesugi, Dakeun Lee, Su Bin Lim

**Affiliations:** 1 Department of Biochemistry & Molecular Biology, Ajou University School of Medicine, Suwon, 16499 South Korea; 2 Department of Biomedical Sciences, Graduate School of Ajou University, Suwon, 16499 Korea; 3 BK21 R&E Initiative for Advanced Precision Medicine, Department of Biomedical Sciences, Graduate School of Ajou University, Suwon, 16499 South Korea; 4 Department of Pathology, Ajou University School of Medicine, Suwon, Republic of Korea

**Keywords:** Transcriptomics, Data integration, Gastrointestinal cancer, Bioinformatics, Gene expression

## Abstract

Gastrointestinal cancer is one of the most burdensome health threats worldwide, accounting for approximately one-quarter of all cancer incidences. Gastric and colorectal cancers are among the most prevalent and lethal malignancies of the gastrointestinal tract globally. Despite steadily rising incidence, the complex cellular landscape comprising the tumor microenvironment and accelerating tumorigenesis remains insufficiently explored. Although single-cell transcriptomics has been employed to investigate this complexity, previous studies are limited by the small number of cells analyzed. In this study, we present a comprehensive single-cell transcriptomic atlas comprising 574,532 cells across 70 cell types from 229 human stomach tissues and 479,629 cells across 70 cell types from 220 human colorectal tissues, spanning diverse phenotypes. Data quality and cell type annotations were rigorous validated utilizing multiple computational tools. Additionally, standardized cell type definitions and annotated clinical information facilitate the usability of this dataset, enabling analysis across clinical subtypes and metastatic states. This resource provides a valuable foundation for meta-analyses of gastric and colorectal cancers at single-cell resolution.

## Background & Summary

The stomach and colorectum, which make up the majority of the digestive system, play crucial roles in food storage, distribution, and processing. Digestive diseases remain one of the greatest global health threats, affecting 7.32 billion people and causing 8 million deaths in 2019^1^. Among these, diseases of the stomach and colorectum are particularly dominant. Colorectal cancer (CRC) is the third most prevalent and the second most lethal cancer globally, while gastric cancer (GC) ranks as the fifth most common and the fourth leading cause of cancer-related death, with the highest incidence reported in Eastern Asia. Without major intervention, the incidence and mortality of both cancers are projected to increase by 2040^2–4^. Although anatomically distinct, the stomach and colorectum share a common endodermal origin and exhibit strikingly similar pathological trajectories, particularly in the progression from chronic inflammation to malignancy. They also display overlapping clinical and molecular features, including perineural invasion^5,6^, microsatellite instability^7,8^ (MSI). Consistent with these shared biological characteristics, GC and CRC are frequently treated using comparable therapeutic strategies, such as fluoropyrimidine-based chemotherapy combined with oxaliplatin^9–11^ (e.g., FOLFOX), immune checkpoint inhibition^12,13^ (e.g., PD-l/PD-L1 inhibitors) in selected molecular subtypes, and anti-HER2–targeted therapy^14,15^ in HER2-amplified tumors. Despite these parallels, most studies have investigated GC and CRC in isolation, potentially overlooking conserved pathogenic mechanisms and shared therapeutic vulnerabilities across the gastrointestinal tract. A comparative and integrated investigation of these malignancies is therefore essential to delineate common cellular programs, immune regulatory circuits, and progression-associated features that transcend anatomical boundaries and may inform unified or cross-applicable therapeutic strategies.

Single-cell sequencing technology has surfaced as a revolutionary methodology for precisely exploring and elucidating the anatomical and physiological view of the human body, portraying cellular and molecular dynamics^16^. Additionally, the integration of single-cell RNA-seq (scRNA-seq) data has enabled meta-analyses that can not only uncover rare cell populations or biomarkers inducing inhibition of treatment efficacy and poor prognosis but also investigate unique cell-cell interactions within the tumor microenvironment (TME), potentially identifying novel therapeutic targets^17–19^. For instance, we previously characterized the diverse cellular landscape of immune cells together with cancer cell subtypes, by integrating multiple non-small cell lung cancer (NSCLC) scRNA-seq datasets. Additionally, we revealed dynamic transcriptomic trajectories of cancer cell subtypes over pseudotime, implying how cancer cells may undergo differentiation during NSCLC progression. Beyond cancer studies, data integration has illuminated diverse molecular signatures in neuroscience, particularly when combined with novel sequencing technologies^20^. In our previous study, we delineated precise neuronal and non-neuronal cell types in hypothalamic brain regions using integrated scRNA-seq data^21^. This approach not only enabled the identification of rare cell types but also validated unconventional nuclear Connect-seq (nuConnect-seq) data. These findings underscore the power of scRNA-seq integration to uncover novel insights into tumor or neuroscience biology. However, the field still lacks comprehensive, publicly accessible scRNA-seq atlases mainly of human gastrointestinal tissues, limiting progress in translational research.

**Fig. 1:**
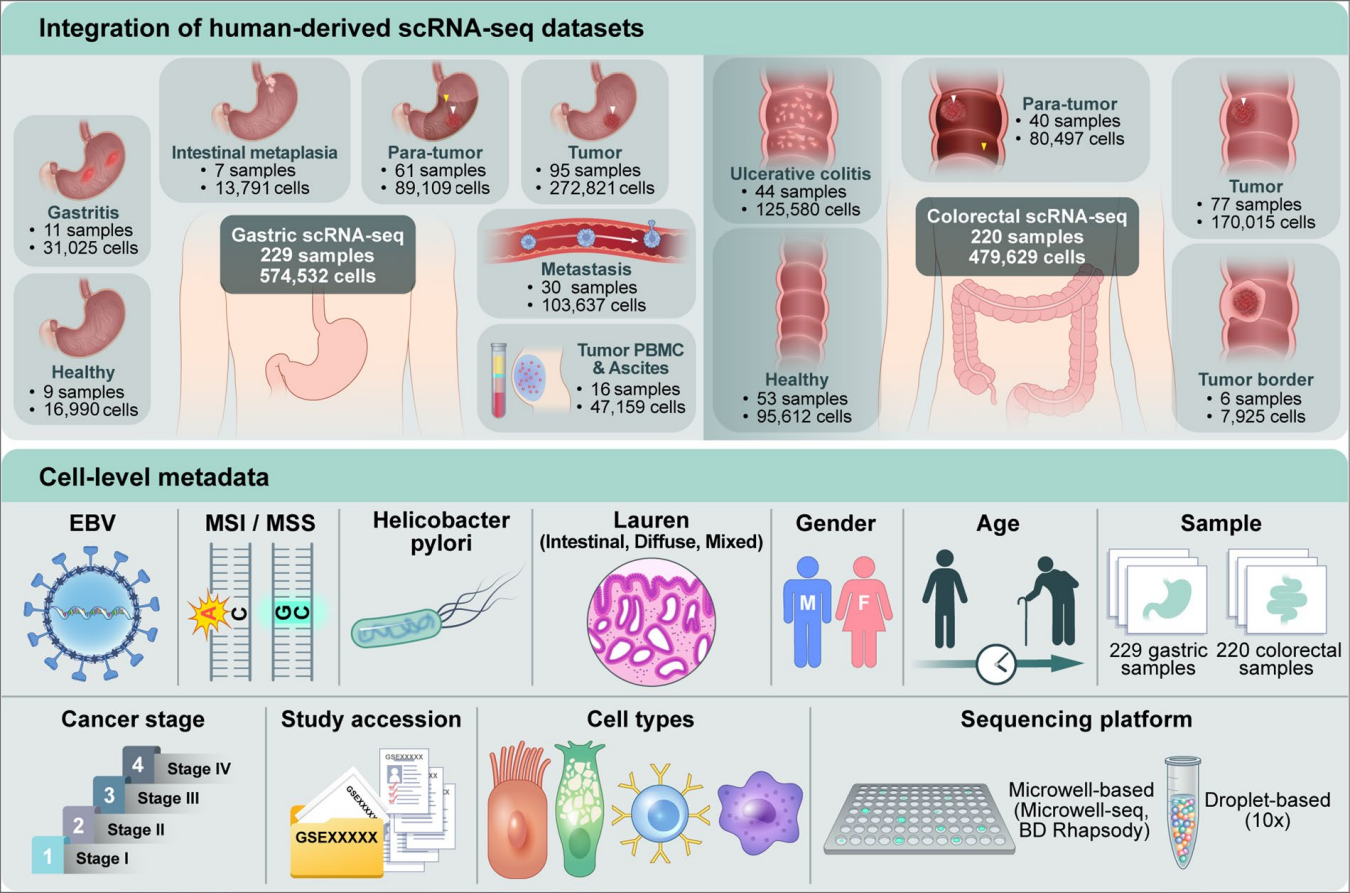
Graphic abstract of dataset.

In this study, we present an extensively curated gastrointestinal scRNA-seq atlas, compiling 20 datasets from 229 gastric and 220 colorectal tissue samples. Through stringent quality control (QC) and robust cell type annotation, 574,532 gastric QC-passed cells and 479,629 colorectal QC-passed cells were acquired, with 70 level 2 cell types identified. The gastric dataset includes 13 distinct phenotypes: normal tissue, three types of gastritis—non-atrophic gastritis (NAG), chronic atrophic gastritis (CAG), and chronic superficial gastritis (CSG)—intestinal metaplasia (IM), para-tumor tissue adjacent to gastric cancer (ADJ_GC), gastric cancer (GC), four distinct metastatic sites—lymph node, liver, ovary, and peritoneal metastasis—as well as peritoneal ascites (GC_PC) and peripheral blood mononuclear cells (GC_PBMC) obtained from gastric cancer patients. The colorectal data are composed of normal, ulcerative colitis (UC), para-tumor tissue adjacent to colorectal cancer (ADJ_CRC), tumor border, and colorectal cancer (CRC). In addition to transcriptomic profiles, the atlas includes detailed clinical metadata such as infection status (e.g., viral or bacterial), histological classification, disease stage and technical variables including cell type annotations and sequencing platform (Fig. 1, Table S1). By providing an integrated single-cell transcriptomic resource that spans both organs across the normal, inflammatory, and malignant continuum, our dataset enables systematic identification of conserved cellular programs underlying gastrointestinal inflammation and tumorigenesis, while also facilitating the characterization of pan-gastrointestinal immune microenvironmental features that may serve as broadly applicable therapeutic targets.

## Methods

### Data collection

Raw count matrices of scRNA-seq were downloaded from the National Center for Biotechnology Information (NCBI) Gene Expression Omnibus (GEO) and Single Cell Portal. To construct the integrated gastrointestinal dataset, we systematically searched for publicly available human scRNA-seq datasets using the following keywords: human stomach scRNA-seq, human colorectum scRNA-seq, gastric cancer scRNA-seq, and colorectal cancer scRNA-seq. Among publicly available human stomach and colorectal scRNA-seq datasets, only those available in raw format and representing diverse cell populations were retained for data integration. Studies focusing solely on individual cell types, such as fibroblasts or immune cells, were omitted. The following datasets^22–32^ were curated to construct the gastric dataset: GSE134520, GSE150290, GSE163558, GSE167297, GSE183904, GSE201347, GSE210347, GSE225275, GSE228598, GSE231539 and GSE234129. For the colorectal dataset, the following datasets^33–40^ were collected: GSE132465, GSE144735, GSE166555, GSE182270, GSE188711, GSE200997, GSE225857, GSE232525 and SCP259.

### Quality control

**Fig. 2:**
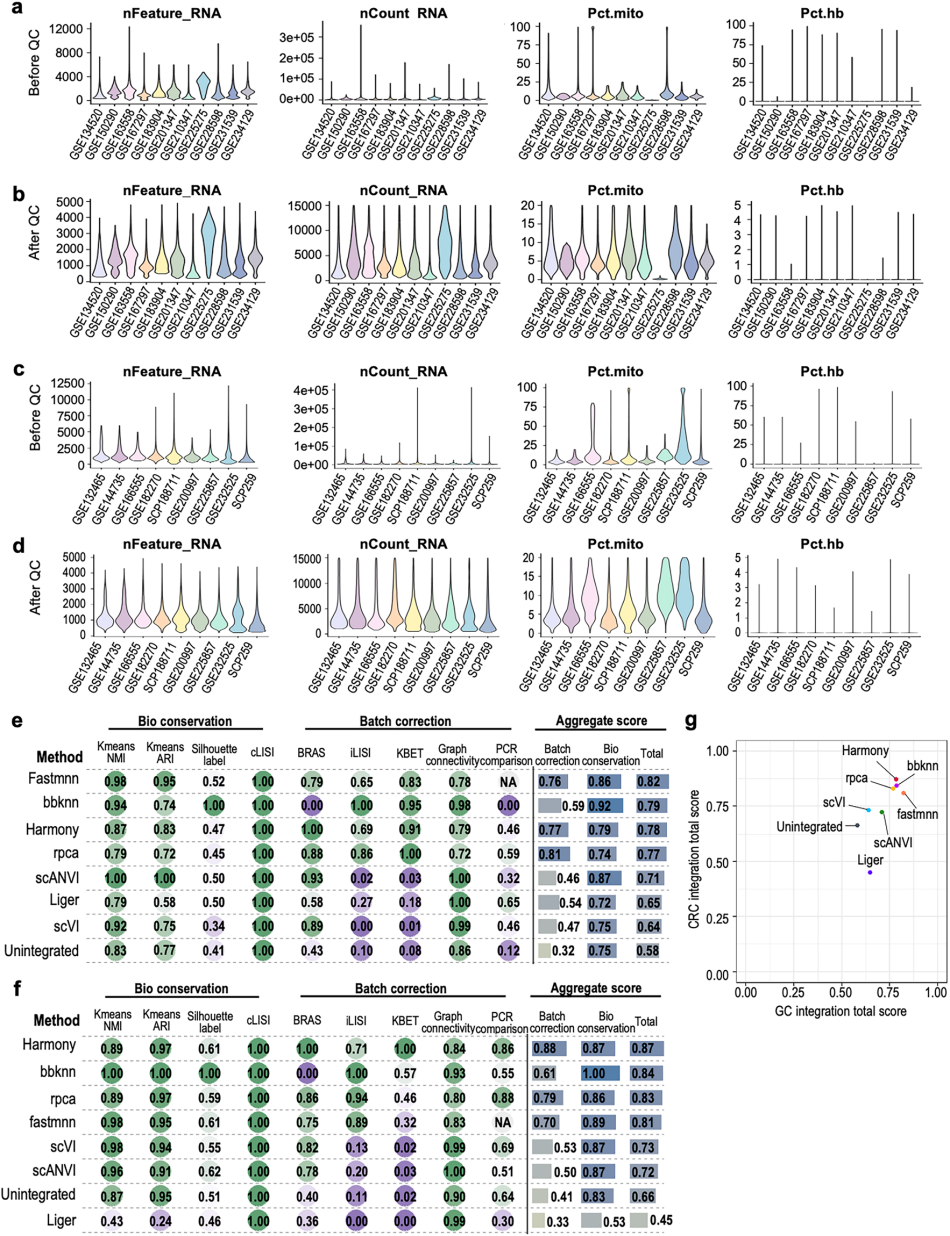
QC and integration metrics. (**a**) Evaluation metrics for gastric scRNA-seq data qualities before and (**b**) after QC. (**c**) Evaluation metrics for colorectal scRNA-seq data qualities before and (**d**) after QC. (**e**) Benchmarking outcome of gastric dataset and (**f**) colorectal data integration. (**g**) Dot plot exhibiting comprehensive integration score of both gastric and colorectal integrated dataset.

Prior to QC, raw data provided in txt, csv, tsv, or mtx formats were imported into R^41^ (v.4.3.3) and converted into Seurat object employing Seurat^42^ (v.5.2.1). These objects were then merged. Low-quality cells were filtered from the merged dataset based on the following criteria: nFeature_RNA > 200, nFeature_RNA < 5,000, nCount_RNA < 15,000, percent_mitochondrial < 20, percent_hemoglobin < 5 (Fig. 2a–d). Genes detected in fewer than three cells across the datasets were discarded. The data were normalized to log scale with a scale factor of 10,000. The top 2,000 variable features were identified using variance stabilizing transformation (VST) algorithm. After data scaling and principal component analysis (PCA), the optimal number of PCs was calculated based on the method proposed by Piper *et al*.^43^. Specifically, PCs were selected based on the minimum value that satisfied two criteria: (1) the value at which individual PCs accounted for only 5% of the standard deviation and the cumulative PCs accounted for 90% of the standard deviation; and (2) the value at which the percentage change in variation between successive PCs was less than 0.1%. Clustering was performed using the shared nearest neighbor with Louvain algorithm. The results were visualized using Uniform Manifold Approximation and Projection (UMAP).

### Data integration and cell type annotation

To opt the most optimal integration methodologies removing batch effect efficiently for our data removing, data integration and benchmarking were performed using seven integration tools under R and python environment, including Harmony^44^ (v1.2.3), bbknnR^45^ (v2.0.2), rliger^46^ (v2.2.1), robust principal component analysis (RPCA) and fastmnn^47^ via IntegrateLayers function in Seurat^42^ (v.5.2.1), scVI^48^ and scANVI^49^ implemented by scvi-colab (v0.14.0). To assess the capacity of robust integration, scib-metrics^50^ (v0.5.8) was utilized for computing integration metrics considering biological conservation and batch correction. Following integration, cell types were rigorously annotated based on established markers and previously published references^32,33,40,51–53^ to ensure consistency and usability across datasets.

### Pseudotime analysis

To improve cell type annotation accuracy, pseudotime analysis was conducted employing Monocle3^54^ (v.1.3.7). Prior to analysis, the Seurat object was converted into a ‘cell_data_set’ object using SeuratWrappers^42^ (v.0.3.2). To support the annotation, cell types were ordered by the median of computed pseudotime values and visualized using ggplot2^55^ (v.3.5.1). For more precise annotation of the highly heterogeneous colorectal CD4T cells, we also applied STCAT^56^ (v1.0.7), an automated T cell subtype annotation tool, alongside traditional marker-based annotation. Cells classified as CD4T cells in the level 2 annotation were extracted and stored in a new Seurat object. This object was then converted into h5ad format using zellkonverter^57^ (v1.16.0). STCAT classified each cell into one of the following subtypes: CD4 Naive, CD4 Tstr, CD4 Tcm, CD4 Tem, CD4 Trm, CD4 Th17, CD4 Trm cell-death, CD4 Tfh, or CD4 Tex. Clusters that could not be annotated were assigned as ‘heterogeneous.’ The resulting subset was converted back to a Seurat object using anndataR^58^ (v0.99.0).

## Data Records

The integrated scRNA-seq datasets, named “Gastric_integrated_data” and “Colorectal_integrated_data”, are available in the figshare repository^59^ in RDS format. Detailed information on data acquisition, dataset description, quality control (QC), and validation procedures is provided on the repository webpage. The availability of raw data and metadata is summarized in Table 1.

**Table 1:** Data description.

GEO accession	Organ	No.QC-passed cells	No.Sample	Platform	Phenotype
GSE134520	Stomach	41,811	13	10x	CAG, NAG, IM, GC
GSE150290	Stomach	16,714	52	10x	CAG, CSG, ADJ_GC, GC
GSE163558	Stomach	40,976	10	10x	ADJ_GC, GC, LN_Meta, LI _Meta, OV_Meta, PC_Meta
GSE167297	Stomach	21,946	14	10x	ADJ_GC, GC
GSE183904	Stomach, Peritoneum	138,401	40	10x	ADJ_GC, GC, PC_Meta, GC_PC
GSE201347	Stomach	98,139	22	10x	ADJ_GC, GC
GSE210347	Stomach	51,220	24	Microwell-seq, 10x	Normal, IM, ADJ_GC, GC
GSE225275	Peritoneum	3,580	4	10x	Normal
GSE228598	Stomach	104,227	28	10x	PC_Meta, GC_PC
GSE231539	Stomach	38,937	5	10x	GC
GSE234129	Stomach	18,581	17	10x	ADJ_GC, GC, LI_Meta, OV_Meta, PC_Meta, GC_PBMC
GSE132465	Colorectum	48,252	18	10x	ADJ_CRC, Border, CRC
GSE144735	Colorectum	23,216	33	10x	ADJ_CRC, CRC
GSE166555	Colorectum	32,250	25	10x	ADJ_CRC, CRC
GSE182270	Colorectum	30,766	9	10x	Normal, UC
GSE188711	Colorectum	23,715	6	10x	CRC
GSE200997	Colorectum	46,717	23	10x	ADJ_CRC, CRC
GSE225857	Colorectum	80,040	16	BD Rhapsody	ADJ_CRC, CRC
GSE232525	Colorectum	4,247	2	10x	CRC
SCP259	Colorectum	190,426	88	10x	Normal, UC

## Technical Validation

### Systematic evaluation of data quality across scRNA-seq datasets

We first assessed the quality of the collected scRNA-seq datasets. As most of the datasets were acquired as raw data, low-quality cells were removed prior to downstream analyses. To ensure consistency across datasets, a standardized QC pipeline, as described in the Methods section, was uniformly applied. We filtered out cells expressing an insufficient or excessive number of detected genes, as well as those exhibiting high percentages of mitochondrial or hemoglobin gene expression (Fig. 2a–d). Following QC, the overall quality of the retained cells was evaluated. In gastric dataset, 86.0% of cells possessed a total count of at least 1,000, while 79.0% maintained a mitochondrial gene fraction below 10% (Fig. 2b). Similarly, in colorectal dataset, 88.3% of cells possessed a total count of at least 1,000, while 76.8% maintained a mitochondrial gene fraction below 10% (Fig. 2d). These metrics collectively validate the robustness of the processed data, ensuring their reliability for all subsequent downstream analyses.

### Benchmarking and selection of data integration methods

To determine the most robust integration approach for our datasets, we evaluated seven commonly used integration methods—Harmony^44^, bbknn^45^, Liger^46^, rpca^42^, fastmnn^47^, scVI^48^ and scANVI^49^—based on their balanced ability to remove batch effect with the preservation of biological signals. Quantitative benchmarking using scib-metrics revealed varying performance across the methods (Fig. 2e,f). Aggregate scores demonstrated that Harmony achieved an optimal balance, maintaining high biological conservation while effectively mitigating technical batch effects. Accordingly, Harmony was selected as the final integration method supported by its superior performance in both gastric and colorectal dataset integration (Fig. 2g, Table S2).

### Assessment of dataset composition

**Fig. 3:**
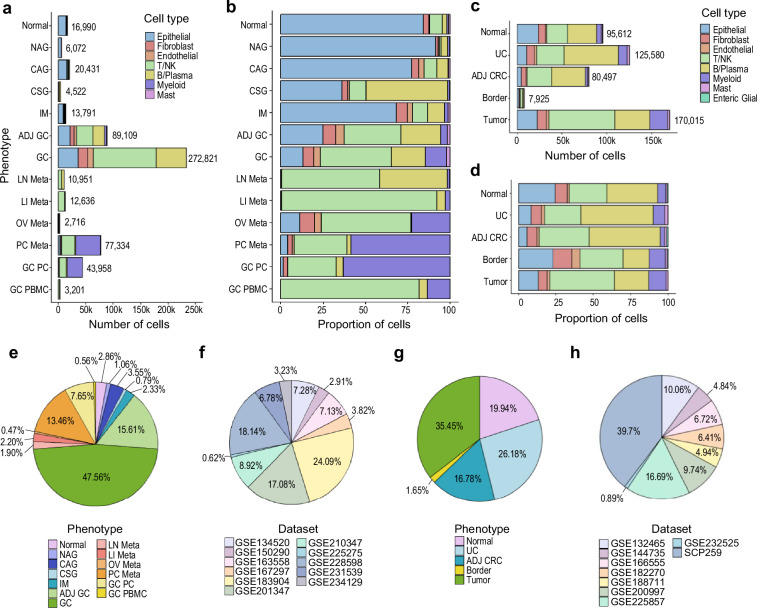
Summary of Data Composition. (**a**) Number of cells and (**b**) proportion of cell types by phenotypes in gastric scRNA-seq data. (**c**) Number of cells and (**d**) proportion of cell types by phenotypes in colorectal scRNA-seq data. (**e**) Pie chart displaying composition of phenotype and (**f**) studies consisting gastric dataset. (**g**) Pie chart depicting composition of phenotype and (**h**) studies consisting colorectal dataset.

To address the potential for representation bias across tissues and patient cohorts, we performed a comprehensive compositional analysis of the integrated data. We first evaluated the distribution of cells across clinical phenotypes to confirm a well-balanced representation of various tissues and disease states (Fig. 3a–d). The integrated datasets encompass a broad spectrum of the disease continuum, spanning normal and inflamed tissues, primary tumors, and diverse metastatic sites. While primary tumor samples constitute the largest proportion of the datasets, reflecting the higher cellular yield obtained from surgical resections compared to biopsies of normal or inflammatory tissues, this dataset also incorporates a substantial number of cells from other phenotypes (Fig. 3e,g).

Furthermore, we examined the contribution of individual datasets to the final integrated data to check for overrepresentation of specific studies (Fig. 3f,h). Cells were derived from multiple independent studies, with no single dataset dominating the overall composition. This balanced representation across phenotypes, conditions, and data sources supports the robustness of the atlas for downstream comparative and integrative analyses.

### Validation of cell type annotation

**Fig. 4:**
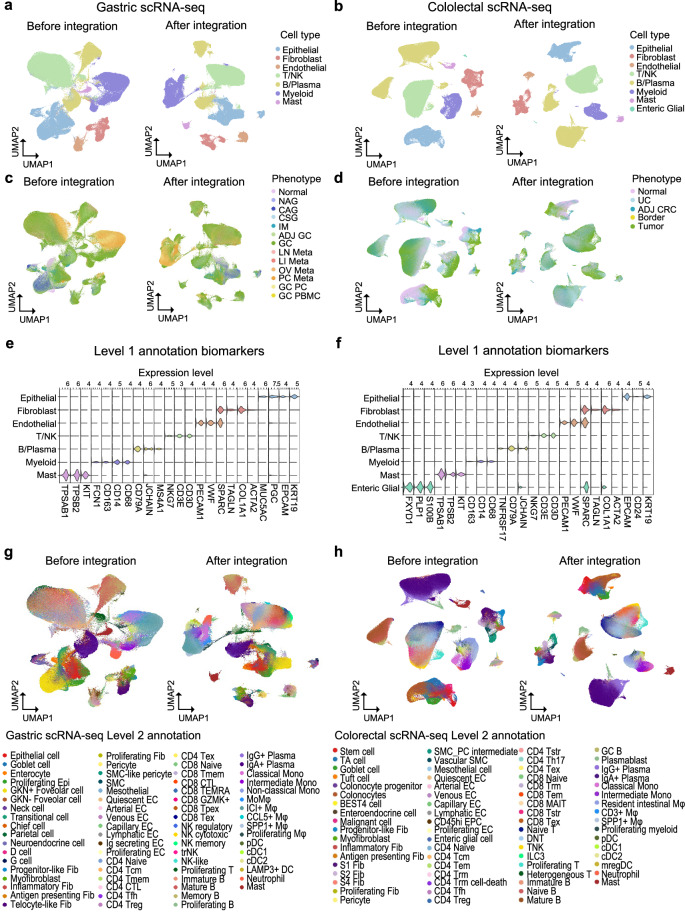
Diverse cellular landscape in stomach and colorectum. (**a**) UMAP displaying level 1 annotation of cell types in stomach and (**b**) colorectum. (**c**) UMAP colored by varying phenotypes in stomach and (**d**) colorectum. (**e**) Violin plot showing expression of well-known marker genes for level 1 cell types in stomach and (**f**) colorectum. (**g**) UMAP exhibiting level 2 annotation of cell types in stomach and (**h**) colorectum.

As shown in Fig. 4a–d and Figure S1a,b, batch effects were successfully eliminated following integration applying the Harmony algorithm. Seven gastric cell types and eight colorectal cell types were designated for level 1 annotation (Fig. 4a,b) based on distinctive expression of well-established marker genes (Epithelial; EPCAM, KRT19, Fibroblast; COL1A1, TAGLN, SPARC, Endothelial; SPARC, VWF, PECAM1, T/NK; CD3D, CD3E, NKG7, B/Plasma; MS4A1, JCHAIN, CD79A, Myeloid; CD68, CD14, FCN1, Mast; KIT, TPSB2, TPSAB1, Enteric Glial; S100B, PLP1, FXYD1) (Fig. 4e,f). 70 cell types were identified for level 2 annotation following re-integration in both stomach and colorectal dataset, respectively (Fig. 4g,h). The level 2 annotations were confirmed by spatial distribution, z-scores of marker gene expression, and cellular composition. The calculated z-scores of marker gene expression is presented in Tables S3-4, while the proportions of cell types across diverse phenotypes are shown in Tables S5-6.

### Validation of non-immune cell type level 2 annotation

**Fig. 5:**
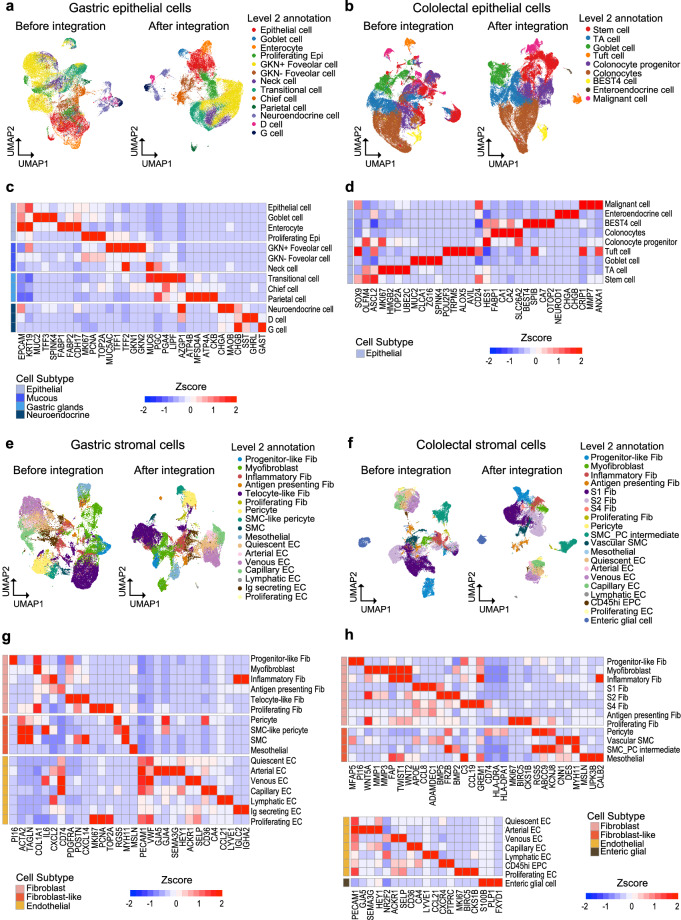
Description of non-immune cell subpopulations. (**a**) UMAP showing level 2 annotation of epithelial lineage cells in stomach and (**b**) colorectum. (**c**) z-score of marker gene expression within epithelial cell types in stomach and (**d**) colorectum. (**e**) UMAP showing level 2 annotation of stromal lineage cells in stomach and (**f**) colorectum. (**g**) z-score of marker gene expression within stromal cell types in stomach and (**h**) colorectum.

Thirteen and nine types of epithelial cells were identified in the stomach and colorectal datasets, respectively, after the removal of artifacts (Fig. 5a,b, Figure S1c-d). The annotations following established markers^51–53,60^ were validated using both gene expression profiles for each cell type (Fig. 5c,d) and their compositions across different phenotypes (Figure S2a-b). Cells expressing markers of both neck cells (MUC6, PGC) and chief cells (PGA4, LIPF, AZGP1) were classified as transitioning cells, consistent with previous studies^61–63^. Notably, epithelial lineages such as goblet cells and enterocytes were dominant in intestinal metaplasia, gastric cancer, and its metastatic samples compared to normal tissue and the three gastritis phenotypes. Colonocytes were identified by the expression of CA1 and SLC26A2^64^. These cells are known to play a critical role in maintaining colonic homeostasis through their ability to shape the colonic microbiota for host benefit^65^. Six fibroblast subtypes, four fibroblasts-like cell subtypes, and seven endothelial cell subtypes were identified in the stomach, whereas eight fibroblast subtypes, four fibroblasts-like cell types, seven endothelial cell types, and one enteric glial cell type were identified in the colorectum (Fig. 5e,f, Figure S1e-f). The expression of stromal cell markers, curated from previous studies^18,40,66–69^, displayed discrete patterns in Fig. 4g,h. In the stomach, PDGFRA+ telocyte-like fibroblasts were observed in considerable abundance in gastritis, metaplasia, and cancer phenotype accord with their reported role in promoting metaplastic and dysplastic environment during gastric carcinogenesis^70^. In the colorectal dataset, three fibroblast subtypes - S1 (APOE, CCL8, ADAMDEC1), S2 (BMP2, BMP5, FRZB) and S4 (C3, CCL19, CD74) - were identified, consistent with prior reports^33,71,72^. A distinct cluster co-expressing pericyte markers (RGS5, ABCC9, KCNJ8) and vascular smooth muscle cell markers (CNN1, DES, MYH11) was annotated as SMC_PC_intermediate, as previously described^33,73^. Mesothelial cells were predominantly enriched in both metastatic and non-metastatic peritoneal phenotypes, in line with proceeded stuides^74,75^, unveiling their capacity to maintain homeostasis including tissue repair in the peritoneum. Pericytes, known contributors to tumor metastasis^76^, were elevated in GC and its metastatic phenotypes compared to other groups. Similarly, the proportion of pericytes was highest in CRC tissue compared to other phenotypes (Figure S2c,d).

### Validation of myeloid cell type level 2 annotation

**Fig. 6:**
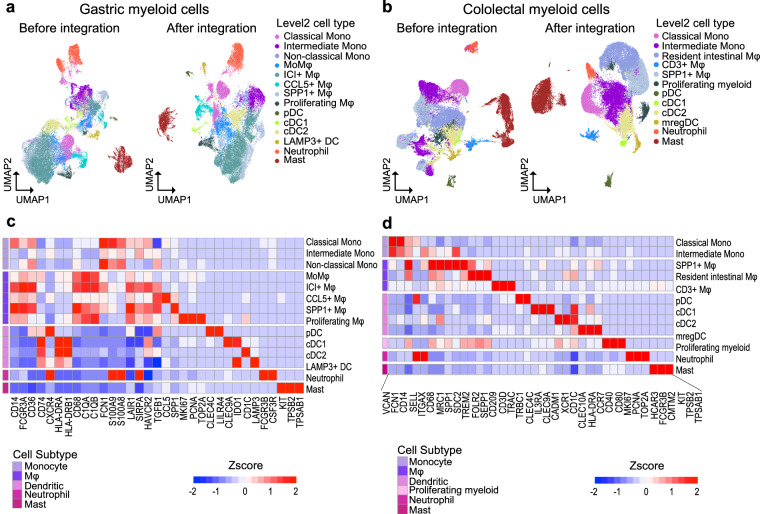
Description of myeloid cell subpopulations. (**a**) UMAP showing level 2 annotation of myeloid lineage cells in stomach and (**b**) colorectum. (**c**) z-score of marker gene expression within myeloid cell types in stomach and (**d**) colorectum.

Employing well-established markers^52,77–81^, 14 and 12 myeloid subtypes were identified in the stomach and colorectum, respectively (Fig. 6a,b, Figure S1g-h). HAVCR2+ macrophages expressing immune checkpoint inhibitor genes such as LAIR1, SIRPA were classified as Immune checkpoint (ICI)+ macrophages. Remarkedly, CCL5+ or SPP1+ macrophages recognized for promoting tumor progression^52,82,83^ were identified in the stomach (Fig. 6c), exhibiting raised proportions in gastric cancer and metastatic phenotypes. Simultaneously, a loss of mast cells was detected during tumorigenesis and metastasis in the stomach (Figure S3a). In the colorectal dataset, resident intestinal macrophages^80^ and SPP1+ macrophages were observed (Fig. 6d). While normal tissue was highly enriched with resident intestinal macrophages, which are thought to play a crucial role in tissue homeostasis^84^, tumor tissue showed a significant enrichment of SPP1+ macrophages. Interestingly, neutrophils were also abundantly present in CRC tissues (Figure S3b). Neutrophils are known to be indirectly recruited by Interleukin-17A (IL-17A) and IL-17F, two key cytokines secreted by T helper 17 (Th17) cells^85^. Indeed, Th17 cells were found to be more abundant in CRC tissue compared to other phenotypes (Figure S3d).

### Validation of lymphoid cell type level 2 annotation

**Fig. 7:**
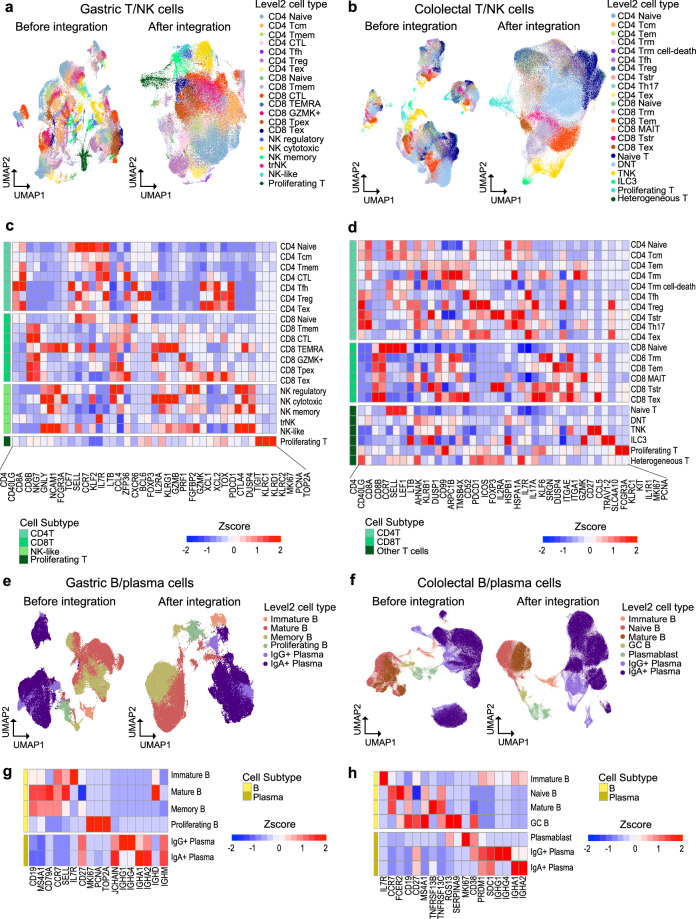
Description of lymphoid subpopulations. (**a**) UMAP showing level 2 annotation of T/NK lineage cells in stomach and (**b**) colorectum. (**c**) z-score of marker gene expression within T/NK cell types in stomach and (**d**) colorectum. (**e**) UMAP showing level 2 annotation of B/plasma cell lineages in the stomach and (**f**) colorectum. (**g**) z-score of marker gene expression within B/plasma cell types in stomach and (**h**) colorectum.

In total, 20 and 22 subtypes of T cells were discerned in gastric and colorectal tissues, respectively, using a curated set of reference markers^32,86–88^ (Fig. 7a,b, Figure S1k-l). To pursue accurate and detailed annotation of T cell subpopulations, we referred to the comprehensive human T cell reference atlas established by Andreatta *et al*.^89^. BCL6+ CD4T cells were assigned as CD4 T follicular helper cells^90^ whereas CXCR6+ CD4T cells were designated to CD4 cytotoxic T cells^91^ in stomach (Fig. 7c). For colorectal CD4T cells, STCAT^56^, an automated T cell type annotation tool, was employed to further refine cell type classification. A total of 45,505 cells were categorized in the following subtypes: CD4 Naive, CD4 Tcm, CD4 Tem, CD4 Trm, CD4 Trm cell-death, CD4 Tfh, CD4 Tstr, CD4 Th17, and CD4 Tex (Fig. 7d). Notably, stomach GZMK+ CD8T cells have previously been associated with poor clinical outcome or recurrence in several diseases^92,93^. In our data, CD8 exhausted T cells were more abundant in para-tumor, tumor and metastasis phenotypes (Figure S3c). Similarly, in CRC tissues, the proportions of stress-response T cells and exhausted T cells were significantly higher compared to other phenotypes (Figure S3d). Applying canonical markers, six stomach B and plasma cell lineages were identified in the stomach and seven in the colorectum (Fig. 7e,f, Figure S1i-j), and their identities were validated by characteristic expression patterns (Fig. 7g,h). Intriguingly, the proportion of IgA+ plasma cells (IGHA1, IGHA2), which can defend gut mucosa against microbiota^94^ were sharply declined in para-tumor, tumor, and metastatic phenotypes in agreement with previous findings^32^ (Figure S3e). In colorectal B cells, a significant increase in IgG+ plasma cells (IGHG1, IGHG4) was observed in UC contrasted to normal tissue (Figure S3f). This is in line with a previous report showing that IgG+ plasma cells infiltrate inflamed mucosa via CXCR4 and contribute to the pathogenesis of human ulcerative colitis^95^.

### Further verification of T cell annotation

**Fig. 8:**
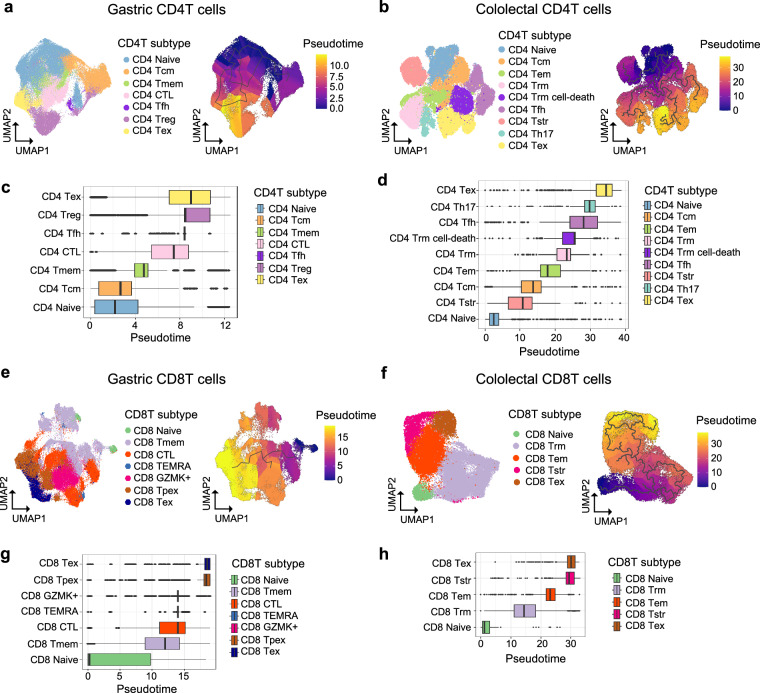
Inference of pseudotime trajectory. (**a**) UMAP showing CD4T cell subtypes (left) computed pseudotime within CD4T cells (right) in stomach and (**b**) colorectum. (**c**) Boxplot describing differentiation of CD4T cells by pseudotime analysis in stomach and (**d**) colorectum. (**e**) UMAP showing CD8T cell subtypes (left) computed pseudotime within CD8T cells (right) in stomach and (**f**) colorectum. (**g**) Boxplot describing differentiation of CD8T cells by pseudotime analysis in stomach and (**h**) colorectum.

Lastly, pseudotime analysis was performed using Monocle3^54^ in order to validate the accuracy of cell type annotation. In stomach CD4T cells, the pseudotime trajectory began from naïve T cells, progressed through memory T cells, and culminated in exhausted T cells resembled with conventional differentiation pathway of CD4T cells (Fig. 8a,c). In colorectal CD4T cells, the pseudotime trajectory also began with naïve T cells and ultimately led to exhausted T cells, reflecting the canonical differentiation trajectory of the CD4T cell lineage (Fig. 8b,d). Stomach CD8T cells disclosed exclusive pattern of trajectory corresponding to recognized CD8T cell’s lineage (Fig. 8e,g). Specifically, stomach CD8 memory cells were sequenced along with memory, cytotoxic, terminally differentiated effector memory T cells. Meanwhile, colorectal CD8T cells paraded a distinct developmental path consistent with the widely recognized model of CD8T cell’s lineage (Fig. 8f,h). These validations confirm the reliability of our cell type annotations and highlight the robustness of the dataset, facilitating downstream analyses in future investigations.

## Supplementary information

Supplementary figures

Supplementary table1. Demographics of tissue donors.

Supplementary table2. Benchmarking of integration

Supplementary table3. Marker gene expression in stomach

Supplementary table4. Marker gene expression in colorectum

Supplementary table5. The proportion of cell subtypes in stomach

Supplementary table6. The proportion of cell subtypes in colorectum

## Data Availability

All datasets analyzed in this study are publicly available under the accession numbers cited in the method section. Final processed and integrated dataset with UMAP embeddings is available at the figshare repository: (10.6084/m9.figshare.c.7843481).

## Code availability

The code used for data integration, pseudotime analysis, and STCAT annotation is available in the figshare repository (10.6084/m9.figshare.c.7843481).
