# Supplementary material for: Integrated single-cell transcriptomic atlas of human gastric and colorectal tissues across diverse phenotypes
Source: Sci Data. 2026 Mar 26;13:751. doi: 10.1038/s41597-026-07108-3 (PMC13184354; doi:10.1038/s41597-026-07108-3)
Supplement: Supplementary file 1 — Supplementary figures [file 41597_2026_7108_MOESM1_ESM.pdf]

Supplementary Figures

Integrated single-cell transcriptomic atlas of human gastric and colorectal tissues across diverse phenotypes

Yunjin Go1,3,† , Aki Uesugi1,2,3,†, Dakeun Lee2,3,4, Su Bin Lim1,2,*

Correspondence to: sblim@ajou.ac.kr

Figure S1. Batch effect correction 2
Figure S2. Non-immune cell subtype composition 4
Figure S3. Immune cell subtype composition 6

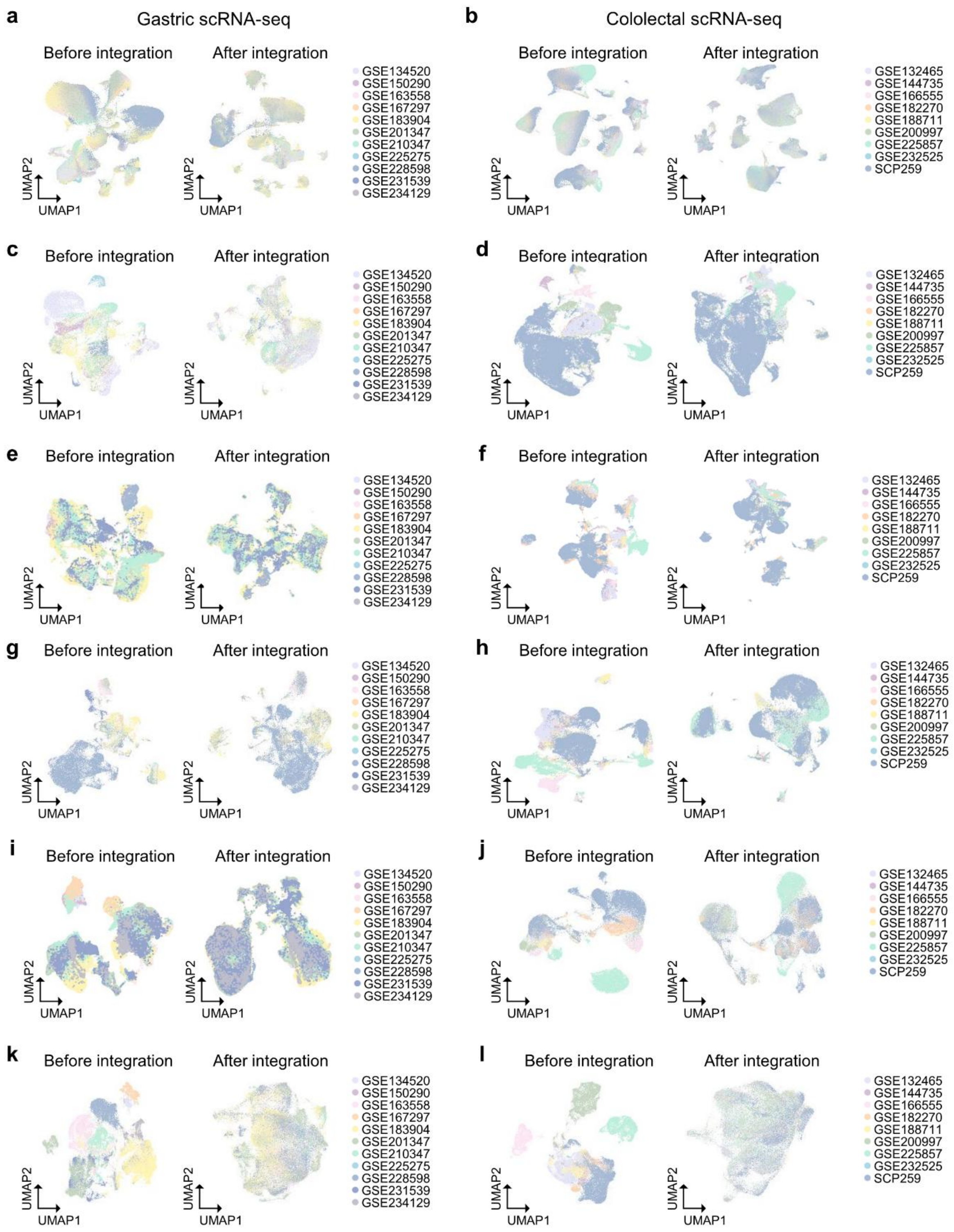

Figure S1. Batch effect correction

(a) Batch effect correction in stomach and **(b)** colorectum scRNA-seq data. **(c)** Batch effect correction in stomach and **(d)** colorectum epithelial cells. **(e)** Batch effect correction in stomach and **(f)** colorectum stromal cells. **(g)** Batch effect

correction in stomach and **(h)** colorectum myeloid cells. **(i)** Batch effect correction in stomach and **(j)** colorectum B/plasma cells. **(k)** Batch effect correction in stomach and **(l)** colorectum T/NK cells.

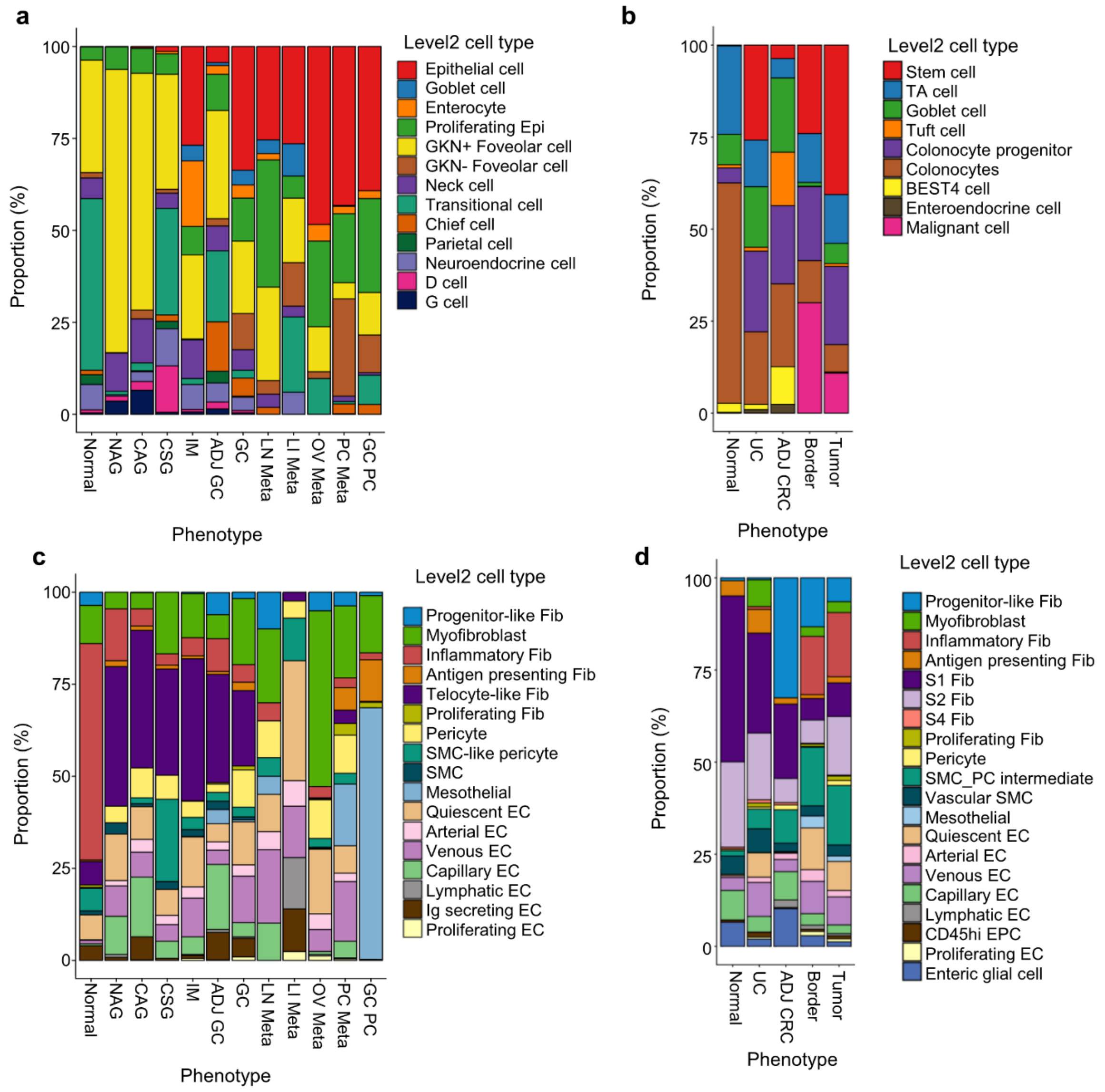

Figure S2. Non-immune cell subtype composition

(a) Proportion of epithelial subtypes among the phenotypes in stomach and **(b)** colorectum. **(c)** Proportion of stromal subtypes among the phenotypes in stomach and **(d)** colorectum.

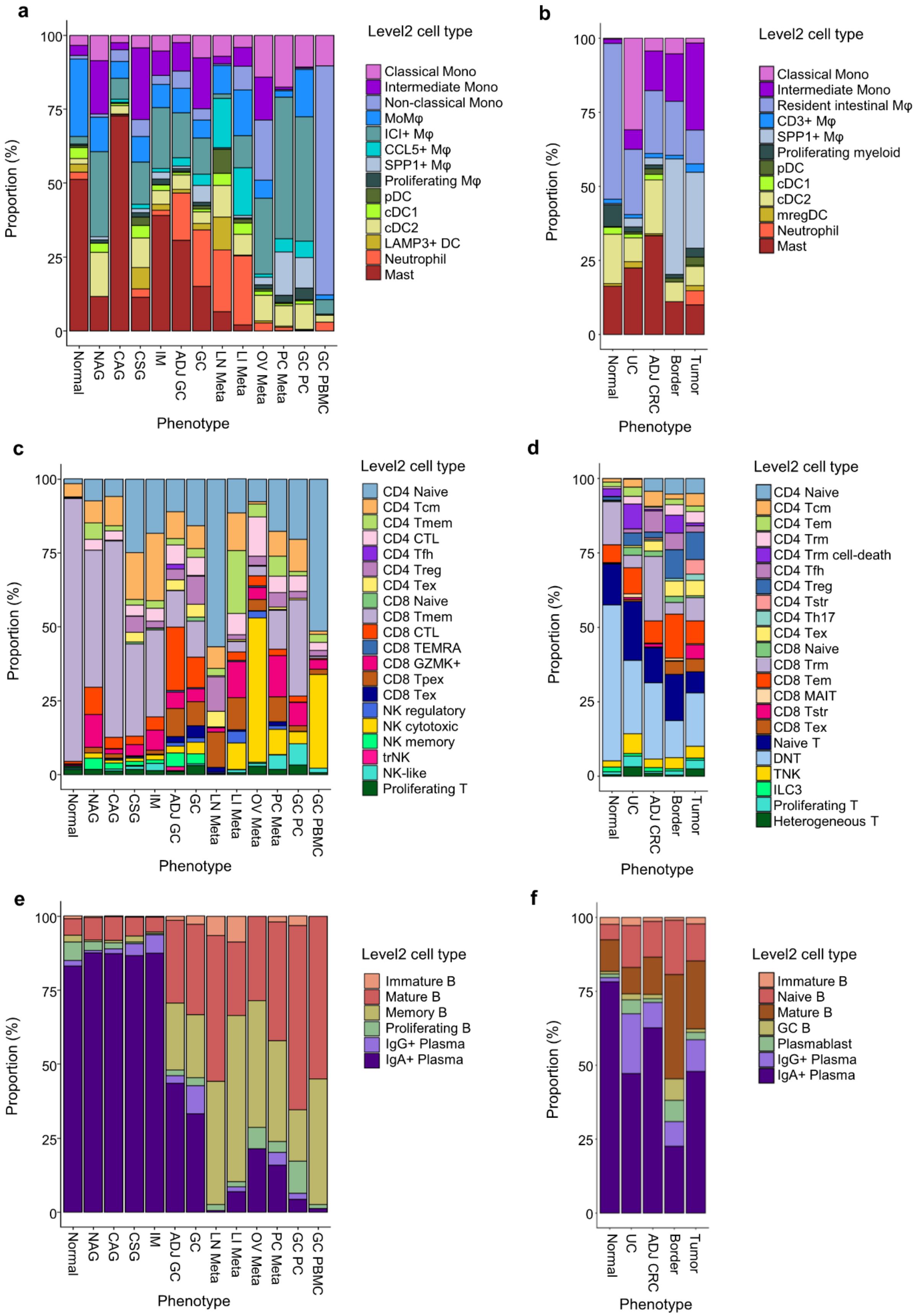

a
Proportion (%)
Phenotype
Normal
NAG
CAG
CSG
IM
ADJ GC
GC
LN Meta
LI Meta
OV Meta
PC Meta
GC PC
GC PBMC
Level2 cell type
Classical Mono
Intermediate Mono
Non-classical Mono
MoMφ
ICI+ Mφ
CCL5+ Mφ
SPP1+ Mφ
Proliferating Mφ
pDC
cDC1
cDC2
LAMP3+ DC
Neutrophil
Mast
b
UC
ADJ CRC
Border
Tumor
Resident intestinal Mφ
CD3+ Mφ
Proliferating myeloid
mregDC
c
CD4 Naive
CD4 Tcm
CD4 Tmem
CD4 CTL
CD4 Tfh
CD4 Treg
CD4 Tex
CD8 Naive
CD8 Tmem
CD8 CTL
CD8 TEMRA
CD8 GZMK+
CD8 Tpex
CD8 Tex
NK regulatory
NK cytotoxic
NK memory
trNK
NK-like
Proliferating T
d
CD4 Tem
CD4 Trm
CD4 Trm cell-death
CD4 Tstr
CD4 Th17
CD8 Trm
CD8 Tem
CD8 MAIT
CD8 Tstr
Naive T
DNT
TNK
ILC3
Heterogeneous T
e
Immature B
Mature B
Memory B
Proliferating B
IgG+ Plasma
IgA+ Plasma
f
Naive B
GC B
Plasmablast

Figure S3. Immune cell subtype composition

(a) Proportion of myeloid subtypes among the phenotypes in stomach and **(b)** colorectum. **(c)** Proportion of T/NK subtypes among the phenotypes in stomach and **(d)** colorectum. **(e)** Proportion of B/plasma subtypes among the phenotypes in stomach and **(f)** colorectum.
